# Cardiac complications associated with COVID-19: a single-center study from Southern Iran

**DOI:** 10.3389/fped.2025.1452353

**Published:** 2025-03-14

**Authors:** Marjan Tariverdi, Mohammadbagher Rahmati, Maryam Mohammadian, Shahrokh Rajaei, Mohammadreza Kargarfard Jahromy, Niloufar Rahimi, Saeed Hosseini Teshnizi, Mohammad Tamaddondar, Shiva Badri, Hossein Abdollahi

**Affiliations:** ^1^ Department of Pediatrics, Clinical Research Development Center of Children’s Hospital, Hormozgan University of Medical Sciences, Bandar Abbas, Iran; ^2^ Department of Pediatrics, Faculty of Medicine, Hormozgan University of Medical Sciences, Bandar Abbas, Iran; ^3^ Department of Pediatric Cardiology, Clinical Research Development Center of Children’s Hospital, Hormozgan University of Medical Sciences, Bandar Abbas, Iran; ^4^ Student Research Committee, Faculty of Medicine, Hormozgan University of Medical Sciences, Bandar Abbas, Iran; ^5^ Department of Biostatistics, Faculty of Nursing and Midwifery, Hormozgan University of Medical Sciences, Bandar Abbas, Iran; ^6^ Department of Nephrology and Internal Medicine, Shahid Mohammadi Hospital, Hormozgan University of Medical Sciences, Bandar Abbas, Iran; ^7^ Community Health Nursing, Clinical Research Development Center of Children’s Hospital, Hormozgan University of Medical Sciences, Bandar Abbas, Iran

**Keywords:** children, COVID-19, heart, echocardiography, cardiac disorder

## Abstract

**Background:**

Children account for a small percentage of COVID-19 cases and tend to exhibit milder symptoms compared to adults. Cardiovascular involvement has been observed in pediatric COVID-19 cases. This study aimed to determine the frequency of cardiac disorders in children hospitalized with COVID-19.

**Methods:**

This cross-sectional study was conducted on pediatric patients admitted to Bandar Abbas Children Hospital, Iran, from March to September 2020. Patients with negative RT-PCR results for SARS-CoV-2, non-COVID-19 pulmonary involvement or pre-existing cardiovascular conditions were excluded. COVID-19 diagnostic subgroups were determined based on national guidelines. Clinical evaluations included chest CT scans to assess pulmonary involvement and cardiac assessments such as clinical symptoms, electrocardiography and echocardiography. Cardiac abnormalities were defined as clinical heart failure, dysrhythmias or abnormal echocardiography. Multivariable logistic regression was applied to analyze the associations between cardiac abnormalities, age and lung involvement, with statistical significance set at *P* < 0.05.

**Results:**

This cross-sectional study was conducted in 2020 on 475 children aged 1 month to 14 years. Among the participants, 48.4% had suspected, 30.5% had probable, and 21.1% had confirmed COVID-19. Cardiac abnormalities were identified in 35.2% of patients, including dysrhythmia (20.2%), heart failure (7.6%), and abnormal echocardiography findings (13.1%). The odds of cardiac abnormalities were 3.3 times higher in children with unilateral lung involvement and 5.9 times higher in those with bilateral lung involvement compared to those without lung involvement. Additionally, older age was associated with a 5.7% reduction in the odds of cardiac abnormalities.

**Conclusions:**

Cardiac abnormalities in pediatric COVID-19 patients show a significant correlation with pulmonary involvement, highlighting their link to disease severity. Routine cardiac assessments may help identify complications and guide management, especially during sporadic cases and seasonal outbreaks.

## Introduction

Since its emergence as an outbreak of atypical pneumonia in Wuhan, China, in December 2019, coronavirus disease 2019 (COVID-19) has rapidly spread worldwide (1), prompting the World Health Organization (WHO) to declare it a pandemic in March 2020 (2). COVID-19 is caused by severe acute respiratory syndrome coronavirus 2 (SARS-CoV-2) (1). Substantial evidence indicates that children account for a smaller proportion of COVID-19 cases compared to adults (3). Most children present with mild symptoms; however, infants have been reported to be at a higher risk of critical illness compared to older children (4). One proposed explanation for the milder presentations in children is the presence of fewer functional angiotensin-converting enzyme 2 (ACE2) receptors, which SARS-CoV-2 uses for cellular entry. Nevertheless, this hypothesis remains inconclusive (5). Although hospital admission and mortality rates are lower in children with COVID-19, severe disease manifestations, including cardiovascular injury, can occur in a minority of cases (6, 7). The underlying mechanisms of cardiac involvement in COVID-19 include myocardial ischemia due to hypoxia, endothelial overactivation, microvascular thrombi from a hyperinflammatory response, and direct myocardial damage caused by SARS-CoV-2 entering cardiomyocytes via ACE2 receptors (8–10).

Myocarditis, pericardial effusion, coronary thrombosis, and other electrocardiographic and echocardiographic abnormalities have been reported as instances of cardiac involvement in COVID-19 (11). The most serious complication in children with COVID-19 is multisystem inflammatory syndrome (MIS-C), which involves cardiovascular manifestations in 80%–100% of affected children (12, 13). Consequently, most studies examining cardiovascular manifestations of COVID-19 in children focus on those with MIS-C. In this study, we aimed to describe and analyze cardiac involvement in a cohort of children hospitalized with suspicious, probable, or confirmed COVID-19, focusing on heart failure symptoms, cardiac dysrhythmias and abnormal echocardiography findings.

## Methods

### Study population

This cross-sectional study was performed on 475 consecutive children aged 1 month to 14 years with suspicious, probable, or confirmed COVID-19 disease admitted to Bandar Abbas Children Hospital, Bandar Abbas, Iran, from March 20, 2020, to September 21, 2020. Children with at least two negative reverse transcriptase-polymerase chain reaction (RT-PCR) results for SARS-CoV-2 virus, 24 h apart, any evidences of pulmonary involvements other than COVID-19 in chest radiography or CT scanning and those with any evidence of underlying cardiovascular disorders such as congenital heart diseases before exposing COVID-19 were all excluded from the study.

In this study, based on the Iranian National Guideline for the Diagnosis and Treatment of COVID-19 in Children, three diagnostic subgroups for pediatric COVID-19 cases were included in the study:

#### Suspicious COVID-19 cases

This group includes children with acute respiratory illness characterized by fever and at least one symptom of respiratory disease such as cough or shortness of breath combined with a history of travel to or residence in areas with reported community transmission of COVID-19 within 14 days prior to symptom onset. It also encompasses children presenting with acute respiratory illness who have had close contact with a confirmed or probable COVID-19 case during the 14 days before symptom onset. Additionally, children with severe acute respiratory illness defined as fever, at least one respiratory symptom and hospitalization are included when no alternative diagnosis thoroughly explains the clinical presentation.

#### Probable COVID-19 cases

This category includes children initially classified as suspicious COVID-19 cases who meet additional diagnostic criteria indicative of COVID-19. This includes children with inconclusive results from COVID-19 testing (e.g., indeterminate RT-PCR findings) or cases where testing could not be performed for any reason. It also encompasses children with pneumonia who exhibit an inadequate clinical response despite proper treatment and whose condition deteriorates rapidly, or who die unexpectedly. Additionally, cases with imaging findings strongly suggestive of COVID-19, based on radiological assessments, are included in this subgroup.

#### Confirmed COVID-19 cases

This group includes children with laboratory-confirmed COVID-19 infection, as evidenced by a positive SARS-CoV-2 RT-PCR test, regardless of clinical signs or symptoms (14).

### Study assessments

The study received ethics approval from the Ethics Committee of Hormozgan University of Medical Sciences under the ethics code IR.HUMS.REC.1400.005 and complies with the statements of the Declaration of Helsinki. The study's retrospective design waived the requirement to obtain informed consent from the parents/guardians of the participants. Baseline characteristics of the children, including demographics (gender, age) and medical history, were extracted from patients’ hospital records. Data on clinical assessments and investigations were retrospectively analyzed as part of this study. During the early stages of the COVID-19 pandemic, clinical protocols for suspected pediatric COVID-19 cases mandated comprehensive evaluations, including chest CT scans and cardiac assessments. Accordingly, all children underwent initial evaluation by CT scan using a Philips V2.0 CT scanner (Philips Medical Systems, Netherlands) to assess the degree of pulmonary involvement.

Cardiac assessments were conducted as part of routine clinical practice and included evaluating clinical manifestations of cardiac dysfunction or failure (e.g., chest pain, trouble breathing, excessive sweating while feeding or exercising), electrocardiography (to detect cardiac dysrhythmias), and 2D echocardiography (to rule out or confirm structural or functional heart abnormalities) using the Vivid™ device (General Electric Company, United States). Cardiac abnormalities were defined as the presence of one or more of the following: clinical manifestations of heart failure, cardiac dysrhythmias or abnormal echocardiography findings.

### Data analysis

Data analysis was performed using the (SPSS) software, version 21.0 (IBM Corp., Armonk, NY, USA). Continuous variables were described using mean ± standard deviation, while categorical variables were reported as frequencies and percentages. Multivariable logistic regression analysis was applied to examine the association between COVID-19 probability and cardiac abnormalities, considering baseline parameters such as age and lung involvement. Odds ratios (OR) and their corresponding 95% confidence intervals (CI) were calculated to interpret the strength and direction of associations. A significance level of *P* < 0.05 was used for all statistical tests (15).

## Results

Of the 475 pediatric patients evaluated in this study, with a mean age of 50.39 ± 47.71 months, 243 (51.2%) were male, and 232 (48.8%) were female. COVID-19 was suspicious in 230 (48.4%), probable in 145 (30.5%), and confirmed in 100 (21.1%) of these children. Overall, cardiac abnormalities were found in 167 (35.2%), including dysrhythmia in 96 (20.2%), heart failure in 36 (7.6%), abnormal echocardiography in 62 (13.1%), and elevated troponin in 101 (21.3%) patients (Table 1). In multivariable logistic regression analysis, confirmed COVID-19 was associated with a 69.3% reduction in the odds of cardiac abnormalities compared to suspected COVID-19 cases (OR = 0.31, 95% confidence interval: 0.12–1.01). Moreover, the odds of cardiac abnormalities were 3.3 and 5.9 times higher in children with unilateral and bilateral lung involvement than in those without lung involvement. Also, older age decreased the odds of cardiac abnormalities by 5.7% (OR = 0.94, 95% confidence interval: 0.89; 1.00) (Table 2). However, COVID-19 probability was not linked to dysrhythmia, heart failure, or abnormal echocardiography.

**Table 1:** Demographics and general findings of the study.

Variables	Results
Demographics (*n* = 475)	
Age (months), mean (SD)	50.39 (47.71)
Sex, *N* (%)	
Male	243 (51.2)
Female	232 (48.8)
COVID-19, *N* (%)	
Suspicious	230 (48.4)
Probable	145 (30.5)
Confirmed	100 (21.1)
Cardiac abnormalities	167 (35.2)
Dysrhythmia	96 (20.2)
Heart failure	36 (7.6)
Abnormal echocardiography	62 (13.1)
Pulmonary abnormalities, *N* (%)	
Abnormal CT	176 (37.1)
Lung involvement	
Unilateral	60 (12.6)
Bilateral	116 (24.4)

N, number; SD, standard deviation; COVID-19, coronavirus disease 2019; CT, computed tomography.

**Table 2:** Association of COVID-19 probability with cardiac abnormalities using logistic regression.

Variables	Univariate analysis		Multivariate analysis	
	OR (95% CI)	*P*-value	OR (95% CI)	*P*-value
Cardiac abnormalities				
Age, years	0.95 (0.90; 1.00)	**0**.**048**	0.94 (0.89; 1.00)	**0**.**036**
Covid-19				
Suspicious	1.00 (ref)		1.00 (ref)	
Probable	1.63 (1.063; 2.49)	**0**.**025**	0.35 (0.12; 1.01)	0.052
Confirmed	0.58 (0.34; 1.00)	**0**.**049**	0.31 (0.15; 0.63)	**0**.**001**
Lung involvement				
None	1.00 (ref)		1.00 (ref)	
Unilateral	1.41 (0.79; 2.52)	0.246	3.32 (1.092; 10.11)	0.034
Bilateral	2.44 (1.57; 3.79)	**<0**.**001**	5.86 (2.203; 15.58)	**<0**.**001**
Dysrhythmia				
Covid-19				
Suspicious	1.00 (ref)		1.00 (ref)	
Probable	1.94 (1.181; 3.177)	**0**.**009**	0.42 (0.122; 1.43)	0.163
Confirmed	0.77 (0.400; 1.496)	0.445	0.41 (0.165; 1.02)	0.055
Lung involvement				
None	1.00 (ref)		1.00 (ref)	
Unilateral	2.42 (1.28; 4.57)	**0**.**007**	4.86 (1.38; 17.02)	**0**.**014**
Bilateral	2.24 (1.34; 3.75)	**0**.**002**	4.60 (1.47; 14.34)	**0**.**009**
Heart Failure				
Sex, female	1.71 (0.86; 3.44)	0.129	1.83 (0.901; 3.714)	0.095
Covid-19				
Suspicious	1.00 (ref)		1.00 (ref)	
Probable	2.16 (1.06; 4.40)	**0**.**034**	1.24 (0.08; 18.71)	0.877
Confirmed	0.29 (0.07; 1.30)	0.107	0.24 (0.04; 1.59)	0.137
Lung involvement				
None	1.00 (ref)		1.00 (ref)	
Unilateral	2.34 (0.92; 5.95)	0.075	1.76 (0.11; 27.36)	0.686
Bilateral	2.23 (1.04; 4.80)	**0**.**040**	1.90 (0.14; 26.42)	0.634
Abnormal echocardiography				
Sex, female	1.42 (0.83; 2.43)	0.200	1.49 (0.86; 2.60)	0.157
Covid-19				
Suspicious	1.00 (ref)		1.00 (ref)	
Probable	2.26 (1.24; 4.13)	**0**.**008**	0.30 (0.08; 1.24)	0.097
Confirmed	1.29 (0.61; 2.72)	0.505	0.51 (0.17; 1.58)	0.242
Lung involvement				
None	1.00 (ref)		1.00 (ref)	
Unilateral	3.03 (1.45; 6.342)	**0**.**003**	8.76 (2.12; 36.13)	**0**.**003**
Bilateral	2.86 (1.56; 5.251)	**0**.**001**	7.32 (2.02; 26.54)	**0**.**002**

Boldface indicates statistical significance (*p* < 0.05). N, number; COVD-19, coronavirus disease 2019; OR, odds ratio.

## Discussion

In the current study, cardiac abnormalities were found in approximately one-third of affected children, dysrhythmia in 20.2%. 13.1% of children suffered from abnormal echocardiography, 7.6% had evidences of acute heart failure. cardiac damages following COVID-19 infection in children may be manifested by a wide range of cardiac defects such as arrhythmias, myocarditis, heart failure even cardiomyopathy. In a multicenter study by Cantarutti et al. (16), cardiac involvement was reported in 29% of children with SARS-CoV-2 infection;. Another study that evaluated cardiac abnormalities associated with COVID-19 in children and young adults, using a multimodality approach, including cardiac computed tomography (CT), cardiac magnetic resonance imaging (CMR) and three-dimensional echocardiography, reported abnormal tissue Doppler indices and strain in almost all patients (17). Minocha et al. showed at least one cardiac abnormality in 73% of children with COVID-19 (18).

Regarding initial clinical manifestations of cardiac dysfunction in affected children include palpitations, dyspnea, chest pain, tightness, conjunctival effusion, polymorphic skin rash and hypotension that if cardiac dysfunction continues and worsens, it will lead to cardiogenic shock, reduced cardiac function capacity, and ultimately acute heart failure (18–20). Also, according to the literature, acute myocardial injury in children suffering COVID-19 is frequently accompanied with raised serum levels of troponin I/T, creatine kinase MB and/or lactate dehydrogenase that increasing the level of troponin I. In the current study, 20.2% of patients were found to have dysrhythmia. A systematic review (9) reported an overall rate of electrocardiogram anomalies at 5.3%, suggesting a higher prevalence of such abnormalities in our study population. Literature indicates that electrocardiographic changes associated with childhood COVID-19 commonly include sinus tachycardia, T-wave inversions, ST-segment abnormalities, right-axis deviation, QT interval prolongation, as well as various arrhythmias and conduction disturbances. Among adolescents, tachycardia is the most frequently observed abnormality, followed by bradycardia (19–21). In the current study, echocardiography-based abnormalities were observed in 13.1% of patients, which falls within the wide range of 9% to 75% reported in the literature for children with COVID-19 (14). This variation in prevalence across studies may reflect differences in study populations, disease severity, diagnostic protocols, or the availability and use of advanced imaging techniques.

Another notable finding of the current study was the significant association between cardiac abnormalities and bilateral lung involvement. To the best of our knowledge, this specific correlation has not been previously investigated in pediatric studies. However, existing evidence indicates that cardiac involvement in COVID-19 is strongly linked to disease severity (22, 23), and pulmonary involvement is known to be more extensive in patients with severe or critical disease compared to those with mild to moderate presentations (24). Additionally, Eslami et al. demonstrated that CT-measured cardiac indices can serve as valuable tools for risk stratification in hospitalized COVID-19 patients, providing insights into the relationship between lung involvement, cardiac dysfunction (25).

We observed that older age in children was associated with reduced odds of cardiac abnormalities, while no significant correlation was found between cardiac abnormalities and sex. Interestingly, a recent study by Brizuela et al. (26) reported a higher mortality rate among male children and increased hospitalization rates in this group. However, it remains unclear whether these outcomes were partially attributable to cardiac involvement. Additionally, infants have been consistently identified as being at higher risk for critical illness compared to older children (4), and this heightened vulnerability may include a greater likelihood of cardiac complications. These findings highlight the need for further research to clarify the relationships between age, sex and cardiac involvement in children with COVID-19, as well as to better understand the underlying mechanisms contributing to these differences.

One limitation of the current study was that only children aged ≤14 years were included, which restricts the generalizability of our findings to the broader pediatric population, typically defined as patients under 18 years of age. This age restriction was due to insurance coverage policies in Iran, which only apply to children ≤14 years. Another limitation was the retrospective nature of the study, which precluded determining the exact timing of cardiac evaluations and whether cardiac abnormalities were directly caused by COVID-19 or influenced by medications used during treatment. Additionally, SARS-CoV-2 RT-PCR was not performed in all patients, leading to a potential underestimation of cardiac abnormalities associated with confirmed COVID-19 cases. Lastly, while echocardiography served as an important diagnostic tool in our study, it has lower sensitivity for detecting cardiac lesions compared to cardiac MRI, potentially resulting in an underestimation of the prevalence and severity of cardiac involvement in the study population (13). The absence of cardiac MRI in our study may have led to an underestimation of the prevalence and extent of cardiac abnormalities in our population.

## Conclusions

Based on the results of the current study, Cardiac abnormalities were observed in pediatric patients with COVID-19, indicating notable cardiovascular involvement in this population. These abnormalities demonstrated a significant correlation with the extent of pulmonary involvement, underscoring their potential association with disease severity. Although the COVID-19 pandemic has ended, sporadic cases and seasonal outbreaks continue to occur. Understanding such complications remains crucial for effective management in these instances and for informing future experiences. Therefore, routine cardiac assessments in pediatric COVID-19 cases could be helpful in identifying potential complications and guiding management strategies.

## Data Availability

The raw data supporting the conclusions of this article will be made available by the authors, without undue reservation.
